# Supplementary material for: Extreme Hyperferritinemia: Causes and Prognosis
Source: J Clin Med. 2022 Sep 16;11(18):5438. doi: 10.3390/jcm11185438 (PMC9505036; doi:10.3390/jcm11185438)
Supplement: Supplementary file 1 [file jcm-11-05438-s001.zip › jcm-1913027-supplementary.pdf]

Supplementary Materials

related to

Extreme hyperferritinemia: causes and prognosis

by Maxime Fauter, Sabine Mainbourg, Thomas El Jammal, Arthur Guerber, Zaepfel, Thomas Henry, Mathieu Gerfaud-Valentin, Pascal Sève, and Yvan Jamilloux

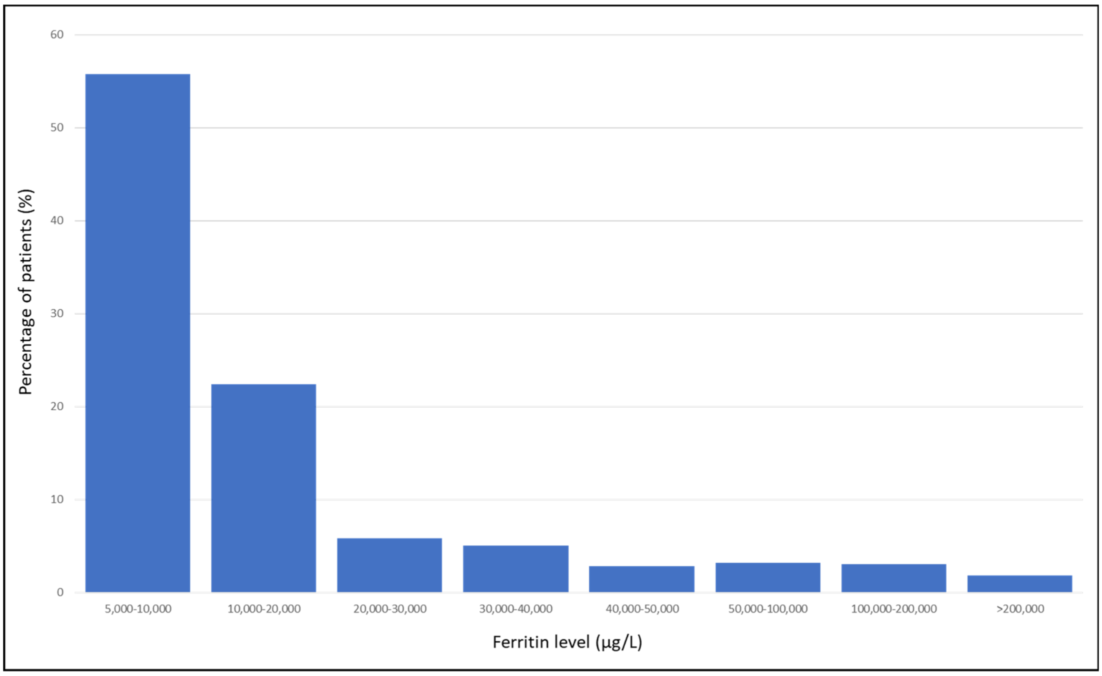

Supplementary Figure S1. Repartition of the patients according to different ferritin levels

Supplementary Table S1. Multivariate analysis by Cox regression model, adjusted for sex, age at diagnosis, ferritin levels and HLH status.

Factor	Hazard Ratio	Lower CI	Upper CI	p-value
Sex	0.93	0.66	1.30	0.664
Age	1.02	1.00	1.03	0.004
Ferritin levels (x10^5)	1.03	1.02	1.04	<0.001
HLH status	1.60	1.07	2.40	0.022
